# Malnutrition prevalence in cancer patients in Belgium: The ONCOCARE study

**DOI:** 10.1007/s00520-024-08324-6

**Published:** 2024-01-27

**Authors:** Marika Rasschaert, Pieter Vandecandelaere, Stéphanie Marechal, Randal D’hondt, Christof Vulsteke, Marie Mailleux, Wendy De Roock, Joanna Van Erps, Ulrike Himpe, Marc De Man, Geertrui Mertens, Dirk Ysebaert

**Affiliations:** 1 Antwerp University Hospital, Antwerp, Belgium; 2 AZ Delta, Roeselare, Belgium; 3 CHC Montlégia, Liège, Belgium; 4 AZ Damiaan, Ostend, Belgium; 5 AZ Maria Middelares, Ghent, Belgium; 6 Clinique Saint-Luc Bouge, Namur, Belgium; 7 Ziekenhuis Oost-Limburg, Genk, Belgium; 8 ASZ Aalst, Aalst, Belgium; 9 Ghent University Hospital, Ghent, Belgium; 10 AZ Sint-Maarten, Mechelen, Belgium; 11 Antwerp University Hospital and Antwerp University, Antwerp, Belgium

**Keywords:** Malnutrition, Advanced cancer, Cachexia, Sarcopenia, Nutritional status, NRS 2002, PROM

## Abstract

**Rationale:**

Unintentional weight loss and malnutrition are common among cancer patients. Malnutrition has been associated with impaired health-related quality of life, less well-tolerated chemotherapy regimens and shorter life duration. In Belgium there is a lack of epidemiological data on malnutrition in oncology patients at advanced stages of the disease.

**Methods:**

Malnutrition assessment data was collected through a prospective, observational study in 328 patients who started a neoadjuvant anticancer therapy regimen or who started 1st, 2nd or 3rd line anticancer therapy for a metastatic cancer via 3 visits according to regular clinical practice (baseline visit (BV) maximum 4 weeks before start therapy, 1st Follow up visit (FUV1) ± 6 weeks after start therapy, FUV2 ± 4 months after start therapy). Malnutrition screening was evaluated using the Nutritional Risk Screening score 2002 (NRS-2002)and the diagnosis of malnutrition by the GLIM criteria. In addition, SARC-F questionnaire and Fearon criteria were used respectively to screen for sarcopenia and cachexia.

**Results:**

Prevalence of malnutrition risk at BV was high: 54.5% of the patients had a NRS ≥ 3 (NRS 2002) and increased during the study period (FUV1: 73.2%, FUV2: 70.1%). Prevalence of malnutrition based on physician subjective assessment (PSA) remained stable over the study period but was much lower compared to NRS results (14.0%—16.5%). At BV, only 10% of the patients got a nutrition plan and 43.9% received ≤ 70% of nutritional needs, percentage increased during FU period (FUV1: 68.4%, FUV2: 67.6%). Prevalence of sarcopenia and cachexia were respectively 12.4% and 38.1% at BV and without significant variation during the study period, but higher than assessed by PSA (11.6% and 6.7% respectively). Figures were also higher compared to PSA. There were modifications in cancer treatment at FUV1 (25.2%) and at FUV2 (50.8%). The main reasons for these modifications at FUV1 were adverse events and tolerability. Patient reported daily questionnaires of food intake showed early nutritional deficits, preceding clinical signs of malnutrition, and therefore can be very useful in the ambulatory setting.

**Conclusions:**

Prevalence of malnutrition and cachexia was high in advanced cancer patients and underestimated by physician assessment. Earlier and rigorous detection of nutritional deficit and adjusted nutritional intake could lead to improved clinical outcomes in cancer patients. Reporting of daily caloric intake by patients was also very helpful with regards to nutritional assessment.

**Supplementary Information:**

The online version contains supplementary material available at 10.1007/s00520-024-08324-6.

## Introduction

Malnutrition in cancer patients is a common problem, with varied prevalence depending on several parameters such as the type and symptomatic burden of the cancer, the history and stage of the disease or comorbidities of the patient. Cancer itself causes anorexia and impaired metabolism, but also cancer treatments such as chemotherapy, targeted therapy and radiotherapy have side effects that contribute to weight loss. Additionally, the physiological changes associated with cancer can lead to malabsorption, diarrhoea, vomiting, etc., contributing to the unintentional weight loss [1]. The systemic inflammation syndrome and the subsequent increase in the production of acute phase proteins result in altered protein turnover, loss of fat and muscle mass, insulin resistance, and impaired glucose tolerance [2]. Sarcopenia occurs as a result of muscle mass depletion and is commonly observed among cancer patients. A systematic review performed in 2017 estimated a 39% prevalence of sarcopenia at cancer diagnosis and associated the presence of pre-therapeutic sarcopenia with increased postoperative complications, chemotherapy-induced toxicity and poor survival in cancer patients [3].

The reported prevalence of malnutrition in recent studies including European patients with cancer ranges from 36% to over 50% [1, 4–7]. One study also identified that more than one third of oncology patients with a significantly decreased oral food intake, did not receive any kind of dietary advice or any prescription of oral supplements, which may contribute to their malnutrition status [1].

ESPEN guidelines state to use a validated screening tool to identify malnutrition in oncological patients in an early stage for instance with the Nutritional Risk Screening (NRS) 2002 [8, 9]. It is the first step towards implementing nutritional interventions to support patients during their disease and to improve disease outcomes, such as survival and patients’ quality of life [8].

Although there is a broad consensus that early recognition of malnutrition is important, there is a lack of implementation in daily clinical practice.

The recent ESMO guidelines [2] recommend the use of the consensus scheme offered by the Global Leadership Initiative in Malnutrition (GLIM) that defines malnutrition by the presence of a positive malnutrition screening test, one phenotypical criterium and one of two etiological criteria [5, 10].

In Belgium there is a lack of epidemiological data on malnutrition in oncology patients and clinical practice concerning screening, assessment and treating malnutrition seems to be very different between several centers.

Our first objective was to prospectively assess malnutrition prevalence according to the NRS 2002 score in patients with confirmed diagnosis of cancer in the need of neoadjuvant anticancer therapy or confirmed diagnosis of metastatic cancer who initiate first, second or third line palliative anticancer therapy in Belgium.

In addition, we wanted to evaluate malnutrition prevalence according to a composite malnutrition score and physician subjective assessment (PSA), to evaluate nutritional practices and clinical outcomes in the defined patient population, and to estimate the percentage of patients with sarcopenia and cachexia. The clinical outcomes for patients receiving an anticancer treatment were also monitored.

## Patients and methods

### Study design

ONCOCARE is a non-interventional prospective multicentric study of solid tumor patients who initiate a neoadjuvant anticancer therapy regimen or who initiate first, second or third line anticancer therapy for a metastatic cancer with palliative purposes.

The Ethics committee of the Antwerp University Hospital approved the study protocol in January 2019 (Belgian Registry nr: B300201837804).

### Patient population

All participants were adult (> 18 years) starting an active oncologic treatment for a confirmed diagnosis of a solid tumor, locally advanced or at metastatic stage. Patient receiving radiotherapy or brachytherapy alone were excluded. All patients provided signed informed consent.

### Objectives and outcomes

The primary study objectives were to assess malnutrition prevalence according to the NRS 2002 score [11] in patients with confirmed diagnosis of cancer in the need of neoadjuvant or metastatic anticancer therapy in Belgium, and to evaluate the diagnosis of malnutrition by according to a composite malnutrition score (GLIM criteria) [10] and physician subjective assessment (PSA), and to estimate the percentage of patients with a risk of sarcopenia and cachexia.

The secondary objective was to evaluate real-world nutritional practices and clinical outcomes in this defined patient population.

### Method

Potentially eligible patients were invited to participate in the study at the baseline visit (BV), which occurred a maximum 4 weeks before initiation of the anticancer therapy regimen.

Once the patient was included, data at BV was collected by the oncologist with sociodemographic data and patient characteristics, cancer related clinical data including the type of oncologic treatment and the nutritional assessment. In addition, the patient collected data was using 2 questionnaires: one done only at baseline to estimate the mean kilocalories (kcal) intake during the three consecutive days before treatment (baseline questionnaire in appendix). A second questionnaire was performed daily to evaluate kcal intake and symptoms related to nutrition and anticancer therapy. A mean of kcal based on the daily questionnaire of the first week after inclusion was used for baseline. (Daily questionnaire in appendix).

**Fig. 1:**
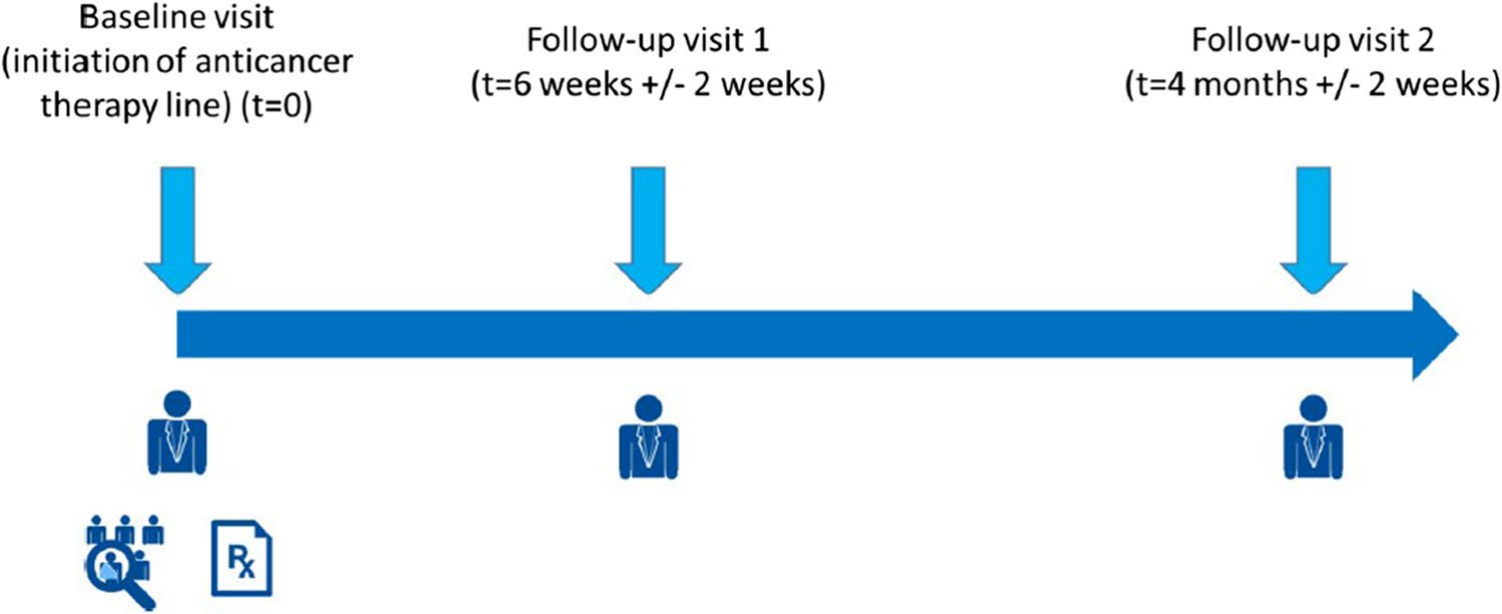
Data collection during study

According to the clinical practice in Belgium, a follow-up visit 1 (FUV1) after 6 weeks (± 2 weeks) and a follow-up visit 2 (FUV2) after 16 weeks (± 2 weeks) was planned, as shown in Fig. 1.

During these FUV the same parameters as baseline were assessed by the oncologist and in addition also treatment related outcomes (treatment toxicity, treatment modification and unexpected hospitalization).

The malnutrition screening was evaluated according to current ESPEN guidelines using the Nutritional Risk Screening score 2002 (NRS-2002) [11] and the diagnosis of malnutrition by the GLIM criteria [10] as primary objective. For the diagnosis of malnutrition by the GLIM criteria it is clear that these cancer patients on chemotherapy/radiotherapy treatment for locally advanced or metastatic cancer fulfilled at least one etiology criterium (presence of inflammation and/or decreased food intake). As phenotypic criterium we used non-volitional weight loss and low BMI.

In our study we also assessed the use of Hand Grip Strength (HGS), as a functional assessment of decreased muscle mass, but this was not used in the GLIM criteria as the HGS is more a supportive measure, rather than a true assessor of decreased muscle mass. The HGS was measured as an indicator of the muscle strength of the upper extremities using a Jamar dynamometer on the dominant hand.

Patient’s nutritional needs were calculated based on ESPEN recommendations targeting 30 kcal/kg actual weight/day.

In addition, SARC-F questionnaire [12] and Fearon criteria [13] were used respectively to screen for sarcopenia and cachexia. The SARC-F questionnaire is a screening tool that can be rapidly implemented by clinicians to identify probable sarcopenic patients. The questionnaire screens patients for self-reported signs suggestive of sarcopenia, which include deficiencies in strength, walking, rising from a chair, climbing stairs, and experiencing falls. Each of the self-reported parameters receives a minimum and maximum score of 0 and 2, respectively, with the greatest maximum SARC-F score being 10. The Fearon criteria comprises a combination of weight loss and/or body mass index (BMI), and skeletal muscle index (determined by CT-imaging) for the diagnosis of cancer cachexia, in which an accurate estimate of weight loss is indispensable. In our study we did not use CT-imaging as it describes real-world observational epidemiological work.

The secondary objective was to evaluate the real-world nutritional practice and clinical outcome in this patient population in oncology wards in Belgium.

### Statistical analyses

The sample size required for the study was calculated to assess the primary objective, to estimate the prevalence of malnutrition in patients receiving anticancer therapy based on NRS-2002. According to recent large European studies [1, 5–7, 13], the prevalence of malnutrition in the cancer population was estimated between 36 and 52%. A prevalence of 50% malnutrition in cancer patients has been used as an approximation for sample size calculation, corresponding to the prevalence requiring the highest sample size. To estimate a prevalence of 50% with a precision of 5%, corresponding to a 95% confidence interval between 45 and 55%, a sample of 385 valid patients is required. This sample was sufficient to assess the prevalence of malnutrition at baseline.

All data analyses were performed using SAS statistics software (SAS Institute Inc., Cary, NC) and in a manner consistent with the Strengthening the Reporting of Observational Studies in Epidemiology (STROBE) guidelines and applicable sections of the Consolidated Standards of Reporting Trials (CONSORT) guidelines. No imputation of missing data was conducted. Patients for whom nutritional intake data collected through the patient diary was not available for > 7 consecutive calendar days, were considered as a drop out. For these patients, data for analysis was only considered until the date of the last available full nutritional intake data.

Univariate analysis was used to assess the relationship between malnutrition status and clinical outcomes or nutritional practice parameters. The statistical significance of the differences in the prevalence of malnutrition was assessed using the Chi-Square test. A logistical multivariate analysis was conducted to identify nutritional practices related to malnutrition, adjusting for potential confounders. Since all statistical tests were only exploratory, the type 1 error will not be adjusted for multiple testing.

## Results

### Patient characteristics

328 patients were included in the study. Table 1 shows the sociodemographic and clinical data of the patients at BV. 298 (90.9%) patients had a FUV1 (6 weeks ± 2 weeks after starting oncologic treatment). 259 patients (86.3%) completed a FUV2 (4 months after starting oncologic treatment). The majority of the patients had stage IV staging at the primary malignancy (75.3%, *n* = 247), stage II and III counted for respectively 11.3% and 10.3% of the included sample. Thirty seven percent (*n* = 91) of the patients had 1 metastatic site at first visit, 30.1% (*n* = 74) had 2 metastatic sites, and 32.9% (*n* = 81) of the patients had 3 or more metastatic sites. Seventy percent of patients (*n* = 229) received 1st line treatment at BV, 18% received 2nd line treatment and about 12% (11.9%, *n* = 39) received 3rd line treatment. At BV, the majority of the patients did belong to the category ECOG-PS 0 (48.2%, *n* = 158) and 1 (47.3%, *n* = 155). The patients belonging to category ECOG-PS 0 declined at FUV1 (6 weeks) to 39.4% (*n* = 117) and further declined at FUV2 (4 months) to 31.8% (*N* = 82).

**Table 1:** Sociodemographic, general and cancer-related clinical data at baseline visit

Variables		Included patients (*n* = 328)
Mean age, years (SD)		63.6 (11,5)
Gender, n (%)	Male	178 (54.3%)
	Female	150 (45.7%)
ECOG-PS, n (%)	0	158 (48.2%)
	1	155 (47.3%)
	2	8 (2.4%)
	3	6 (1.8%)
	4	1 (0.3%)
Primary tumor site, n (%)	Bladder	21 (6.4%)
	Breast	51 (15.5%)
	Upper GI	31 (9.5%)
	Lower GI	66 (20.1%)
	Head and neck	6 (1.8%)
	Hepato/Pancreas/Billiary	29 (8.8%)
	Lung	50 (15.2%)
	Prostate	28 (8.5%)
	Renal	9 (2.7%)
	Ovarium	16 (4.9%)
	Cervix	4 (1.2%)
	Eye- Melanoma	1 (0.3%)
	Brain	1 (0.3%)
	Sarcoma	2 (0.6%)
	Skin	1 (0.3%)
	Endometrium	3 (0.9%)
	Testis	4 (1.2%)
	Eye	1 (0.3%)
	Vulva	1 (0.3%)
	Thymus	1 (0.3%)
	Sarcoma	1 (0.3%)
	Gynecologic (unspecified)	1 (0.3%)
Patients starting neoadjuvant therapy, n (%)		110 (33.5%)
Number of metastatic sites, n (%)	1	91 (37.0%)
	2	74 (30.1%)
	≥ 3	81 (32.9%)
	Missing	82

### Prevalence of (the risk of) malnutrition

Prevalence of the risk of malnutrition at BV, according to NRS 2002, was high: 54.5% of the patients (171 patients over a valid sample of 314) had a NRS > 3, meaning these patients were nutritionally at risk. This prevalence of risk of malnutrition increased after baseline: 73.2% of the patients had a NRS ≥ 3 at FUV1 and 70.1% of the patients at FUV2 were nutritionally at risk (NRS ≥ 3). Based on the GLIM score, 26.3% of the patients had malnutrition at BV with data only available for 224 out of 328 patients included in study sample. The prevalence of malnutrition increased over the study period (53.8% at FUV1, and 61.1% at FUV2). Evolution of GLIM scores during the study period must be interpreted with caution due to the low number of patients having complete information. Considering that a combination of different parameters (weight, height, nutritional need, malabsorption due to gastrointestinal disease and CRP) contribute to the GLIM score, the score could not be calculated if one of these parameters was not available. Prevalence of malnutrition at each visit was underestimated by the physician versus NRS 2002 and GLIM score. Table 2 shows the prevalence of (the risk of) malnutrition according to the NRS 2002 score, the GLIM score and according to the subjective assessment of the physician at each visit.

**Table 2:** Nutritional parameters at baseline visit, follow-up visit 1 and follow-up visit 2

Variable		Baseline Visit	6 weeks visit	4 months visit
BMI (kg/m^2^)	Mean (SD)	26.2 (4.8)	25.9 (4.7)	25.9 (4.7)
Nutritional need (kcal)	Mean (SD)	2,298 (481)	2,295 (480)	2,289 (473)
Caloric intake (total kcal)	Mean (SD)	1,836 (794)	1,315 (715)	1,290 (709)
Caloric intake (kcal/kg BW)	Mean (SD)	24.8 (11.49)	17.8 (10.14)	17.4 (9.76)
Nutritional deficit, % (N), categorical:				
< 50% of nutritional needs	% (n)	16.9% (*n* = 40)	45.9% (*n* = 106)	46.01% (*n* = 98)
50–70% of nutritional needs	% (n)	27.0% (*n* = 64)	22.5% (*n* = 52)	21.6% (*n* = 46)
> 70% of nutritional needs	% (n)	56.1% (*n* = 133)	31.6% (*n* = 73)	32.4% (*n* = 69)
Malnutrition:				
NRS 2002	NRS ≥ 3, % (n)	54.5% (*n* = 171)	73.2% (*n* = 180)	70.1% (*n* = 117)
	Valid n	314	246	167
GLIM	% (n)	26.3% (*n* = 59)	53.8% (*n* = 14)	61.1% (*n* = 11)
	Valid n	224	26	18
Physician Subjective Assessment (PSA)	% (n)	16.5% (*n* = 54)	14.1% (*n* = 42)	14% (*n* = 36)
	Valid n	327	297	257
Sarcopenia:				
SARC-F questionnaire	% (n)	12.4% (*n* = 40)	15.1% (*n* = 43)	15.5% (*n* = 39)
	Valid n	322	285	251
Physician Subjective Assessment	% (n)	11.6% (*n* = 38)	11.1% (*n* = 33)	13.2% (*n* = 34)
	Valid n	328	297	258
Cachexia:				
Fearon criteria	% (n)	38.1% (*n* = 118)	35.8% (*n* = 101)	37.7% (*n* = 92)
	Valid n	310	282	244
Physician Subjective Assessment	% (n)	6.7% (*n* = 22)	6.7% (*n* = 20)	6.6% (*n* = 17)
	Valid n	328	297	258

### Nutritional assessment and intervention

Mean caloric intake decreased from 24.8 kcal/kg BW/day at BV to 17.8 kcal/kg BW/day at FUV1 and 17.4 kcal/kg BW/day at FUV2. Table 2 shows the nutritional intake, target, and balance at each visit. Other nutritional practices are presented in Table 3. At BV, only 10% (9.8%, *n* = 32) of the patients had a nutritional plan, and only 3 patients (0.9%) had a prescription of clinical nutrition (enteral and parenteral nutrition). More than half of the patients had consultation visits to the dietitian or nutritionist between the study visits (52.2% of the patients had consultation visits between BV and FUV1 and 53.5% of the patients had consultation visits between FUV1 and FUV2). These patients visiting a dietitian or nutritionist had on average 2.5 consultation visits between BV and FUV1 and between FUV1 and FUV2. The prescription of clinical nutrition increased slightly over the study period (FUV1: 2.0%, FUV2: 4.2%).

**Table 3:** Nutritional practices at baseline visit, follow-up visit 1 and follow-up visit 2

		Baseline visit (*n* = 328)	6 weeks visit (*n* = 298)	4 months visit (*n* = 259)
Nutritional plan	No	296 (90,2%)		
	Yes	32 (9,8%)		
Use of clinical nutrition	No	325 (99,1%)	292 (98,0%)	248 (95,8%)
	Yes	3 (0,9%)	6 (2,0%)	11 (4,2%)
Type of clinical nutrition	Enteral tube feed	2 (66,7%)	3 (50,0%)	2 (18,2%)
	Parenteral nutrition	1 (33,3%)	3 (50,0%)	9 (81,8%)
Use of oral nutritional supplements	No	300 (91,5%)	237 (79,5%)	203 (79,0%)
	Yes	28 (8,5%)	61 (20,5%)	54 (21,0%)
Consultation to dietitian since last visit	No		142 (47,8%)	120 (46,5%)
	Yes		155 (52,2%)	138 (53.5%)

### Clinical outcomes

The prevalence of sarcopenia and cachexia according to respectively SARC-F questionnaire and Fearon criteria, compared with the PSA, is presented in Table 2. For sarcopenia there is a good correlation between SARC-F questionnaire and PSA, the difference is more important for cachexia with an underestimation by the PSA compared to the Fearon criteria. A univariable analysis was done to assess the relationship between nutritional status at baseline and clinical outcome at follow up visit at 6 weeks (results presented in Table 3). Patients at risk of malnutrition at baseline show a higher frequency of sarcopenia assessed by PSA (6.6% vs 6.6%, *p* = 0.017), higher frequency of cachexia according to Fearon criteria (50.0% vs 19.7%, *p* < 0.001) and according to PSA (12.% vs 1.5%, *p* = 0.004), higher unexpected hospitalization rate (21.1% vs 10.9%, *p* = 0.027), and higher mortality rate (7.6% vs 2.1%, *p* = 0.027) at 6 weeks follow up visit. Similar results were noticed at 4 months follow up visit (data not shown). Multivariable analysis for the presence of cachexia and patient survival was not performed due to low number of patients with each of these conditions.

Table 4 presents the drug modifications and unexpected hospitalizations that occurred at FUV1 and 2. This data showed an increase of drug modification between BV & FUV1 (25% mainly with dose reduction and change in drug regimen), and between FUV1 & 2 (for 50,8% of patients mainly with drug regimen changes at 59,2%). About 95% (94.2%) of the patients presented symptoms and/or toxicities at FUV1. One hundred seventy-nine patients ( 84.8%) experienced fatigue/asthenia, 62.1% had quick satiety, 56.4% had constipation, 55.5% had nausea, half (50.2%) had diarrhoea and 46.9% of the patients had taste changes. Mucositis occurred in 25.6% of the patients and 21.8% of the patients experienced vomiting. Between BV and FUV1, 16.4% of the patients (*n* = 49) had an unexpected hospitalization. Most important reason for these unexpected hospitalizations was adverse events (81.8%).

**Table 4:** Drug modifications and unexpected hospitalizations at follow-up visit 1 and follow-up visit 2

		*6 weeks visit* *(N* = *298)*	*4 months visit* *(N* = *259)*
Modification on the drug treatment regimen since last visit	No	223 (74.8%)	127 (49.2%)
	Yes	75 (25.2%)	131 (50.8%)
	Missing	0	1
Type of modification (1)	Change in drug regimen	39 (45.3%)	93 (59.2%)
	Temporary interruption	25 (29.1%)	46 (29.3%)
	Dose reduction	22 (25.6%)	18 (11.5%)
Reason for change (1)	Not available	1 (1.2%)	3 (1.9%)
	Disease progression	8 (9.3%)	28 (17.8%)
	Adverse event	52 (60.5%)	60 (38.2%)
	Other	25 (29.1%)	66 (42.0%)
Unexpected hospitalization	yes	49 (16.4%)	50 (19.4%)
	no	249 (83.6%)	208 (80.6%)

(1) Percentage reported over the total sample of patients

## Discussion

Our multicenter “real-life” cohort study confirms that in Belgium, as elsewhere, malnutrition remains underdiagnosed in oncological patients [1, 4–7]. At baseline, already more than half of cancer patients, regardless of the line of treatment, were at risk of malnutrition using NRS-2002 and a quarter of patients were malnourished using GLIM criteria as assessment tool. Interestingly, this nutritional status is largely underestimated by the subjective assessment of the treating oncologist, reinforcing the need to use validated and existing tools. It’s noteworthy that tools like NRS-2002 and GLIM, originally validated in hospitalized patients, could also be used to screen and assess malnutrition in our ambulatory oncological day clinic. An early multidisciplinary approach including oncologists, surgeons, specialized nurses and onco-dietitians, adapting nutritional support may impact outcomes with longer overall survival, less treatment modifications or discontinuations, better treatment tolerability with less adverse events and maintenance of quality of life [8].

During the observation period, risk of malnutrition prevalence even increased up to 70% within the first 6 weeks after baseline, while malnutrition was already prevalent up to 50%, showing the need for nutritional support throughout treatment, thereby limiting the risk of cachexia, aligned with literature [14–17]. Indeed, this study could show that the mean caloric intake was already deficient at baseline, decreasing significantly further after 6 weeks. Apparently, only 10% of patients received a nutrition plan at BV, mainly diet enrichment combined with the use of oral nutritional supplements, as first step as recommended in ESPEN guidelines. The use of clinical nutrition was limited (around 1%), with tube feeding or supplementary parenteral feeding hardly being considered. The lack of early nutritional support explains the increase in malnutrition rate, in parallel with a decrease of oral intake between BV and FUV1. It is only later, after 6 weeks and thereafter, that a dietetic consultation is considered with a potential impact on the stabilization of malnutrition rate and nutritional intake changes between FUV1 & 2. This data reinforces the need for early intervention by a dietitian, before any treatment initiation, in order to reduce the nutritional deficits and provide proper nutritional support.

Of course, our study has the well-known drawbacks of other observational studies with some bias and limitations but provides information on current practices in Belgian oncologic centers. ONCOCARE was an observational study, using NRS-2002 and the GLIM score as tools to detect risk of malnutrition and to assess malnutrition. Although the NRS-2002 is not a diagnostic tool, it was much more precise in detecting the risk of malnutrition rather than the PSA. Moreover, due to insufficient data for this observational study of real-world practices, no hard conclusion on the GLIM criteria could be made at this stage, as there were no data on muscle mass. In this study we had to limit the phenotypic GLIMS criteria to weight loss and low BMI. Apparently, the GLIM criteria, as a composite score, seems difficult to realize in daily practice. Nevertheless, the strength of this study is that this multicenter study, largest prospective study in Belgium evaluating the prevalence of malnutrition in cancer patients, as “real-world” data, confirming the findings of other European studies [5–7, 18] and providing an overview of the current clinical nutrition practices for advanced cancer patient treated with an active oncologic therapy. The completed patient daily questionnaire reporting outcomes also added interesting additional information to this study.

There is a significant difference between the prevalence of malnutrition using the screening tools (NRS 2002) and the physician subjective assessment. These results suggest a low detection of oncology patients at risk of malnutrition in terms of clinical practice in Belgium with a delay in starting an effective nutritional support and potentially reducing the negative impact on outcomes. As the physician’s judgement led to underestimation of malnutrition compared to existing tools, there is need for a multidisciplinary team effort to assess the nutritional status as early as possible after the diagnosis of cancer, before starting any treatment and during the treatment trajectory. There is probably a need to rethink the way of resources are allocated between in-hospital and ambulatory setting. In addition, an initiative to involve the patient with an active role in his/her oncological journey seems to be an attractive way to explore. Only then an impact can be expected on patient outcome, treatment modifications and maintenance of quality of life.

Indeed, a real added value to this study was the completed patient daily questionnaires of food intake as a patient reported outcome measurement (PROM) [daily questionnaire in appendix] evaluating nutritional deficits. These deficits, already present at-large during baseline visit, increase significantly in the first 6 weeks, thereby preceding the detection of malnutrition and/or sarcopenia by the existing tools like the NRS-2002, GLIM criteria or the Fearon cachexia criteria. PROMS have already demonstrated clinical efficacy in patients receiving anticancer treatment, however nutritional parameters are currently not included in the cross-cutting set of symptoms (e.g. pain, nausea, vomiting, constipation, diarrhea, dyspnea, insomnia, depression and physical function) as proposed by the ESMO guidelines [19]. If these PROMs can be picked up earlier and regularly by the multidisciplinary team, an early and adaptive intervention could be possible without waiting the scheduled follow-up visit. This could result in more patient empowerment increasing their motivation with an active role and offering a better quality of life with an adequate nutritional support.

## Conclusion

Detecting nutritional deficits early, using PROMs and the existing tools of screening and assessment of malnutrition, and adjusted nutritional intake at initiation of any treatment, could lead to better clinical outcomes in cancer patients. The potential of PROMs in ambulatory oncological setting and nutrition support merits furthers prospective investigations.

### Supplementary information

Below is the link to the electronic supplementary material.

Supplementary file1 (PDF 218 KB)

Supplementary file2 (PDF 222 KB)

## Data Availability

Data are available on request, as they are stored on the repository of IQVIA (https://www.iqvia.com concerning IQVIA ref SFDC1208380) which can accessed on motivated request.
