# Supplementary material for: Malnutrition prevalence in cancer patients in Belgium: The ONCOCARE study
Source: Support Care Cancer. 2024 Jan 27;32(2):135. doi: 10.1007/s00520-024-08324-6 (PMC10821821; doi:10.1007/s00520-024-08324-6)
Supplement: Supplementary file 1 — Supplementary file1 (PDF 218 KB) [file 520_2024_8324_MOESM1_ESM.pdf]

Onderzoeker	Patiëntcode

IMS Health & Quintiles are now IQVIA™

FRESENIUS KABI caring for life

VERTROUWELIJK

DAGELIJKSE VRAGENLIJST VOOR DE PATIËNT

TITEL VAN DE STUDIE: Observationele studie naar ondervoeding bij kankerpatiënten in België die palliatieve of neoadjuvante antikankertherapie krijgen
OncoCare-studie

STUDIECODE (PROTOCOLNR.): NuSt-027-CNI, OncoCare-studie

SPONSOR : Fresenius Kabi Belgium nv/sa

STUDIELOCATIE:

HOOFDONDERZOEKER:

ONDERZOEKERSCODE

AFLEVERDATUM DOOR SITE AAN PATIËNT:
In te vullen door de site
DD MM JJJJ

AFLEVERDATUM DOOR PATIËNT AAN SITE:
In te vullen door de site
DD MM JJJJ

INSTRUCTIES

Bedankt voor uw interesse om deel te nemen aan deze studie!

Zoals besproken met uw arts, rekenen we graag op uw medewerking om deze vragenlijst op dagelijkse basis in te vullen gedurende de komende 4 maanden (of zoals aangegeven door uw arts).

Onthoud dat al uw antwoorden strikt vertrouwelijk worden behandeld. De gegevens die u invult, worden geïdentificeerd aan de hand van een onderzoekers- en patiëntcode rechtsboven iedere bladzijde van de vragenlijst.
De koppeling tussen uw identiteit en deze code is alleen bekend bij het personeel in uw ziekenhuis. Schrijf nergens uw naam of andere traceerbare gegevens (bijv. initialen, geboortedatum enz.) op de vragenlijst.

Breng deze vragenlijst mee naar uw arts bij al uw vervolgafspraken gedurende uw deelname aan het onderzoek. In sommige gevallen ontvangt u een nieuw exemplaar van de vragenlijst tijdens uw afspraken. Het ziekenhuispersoneel zal uitleggen hoe u deze nieuwe vragenlijst moet invullen.

Lees de vragen zorgvuldig door en bespreek eventuele twijfels en zorgen met uw arts of ander gemachtigd personeel (bijv. verpleegkundige, diëtist).

We adviseren u de vragenlijst ‘s avonds in te vullen, zodat u de informatie voor die dag volledig kunt noteren.

Om de vragenlijst in te vullen, volgt u de onderstaande stappen:

1. Vul de datum van de huidige dag in.
2. Kruis het vakje op de schaal aan dat uw eetlust het meest accuraat weergeeft op de huidige dag (op een schaal van 1 tot 10 waarbij '1' staat voor geen eetlust en '10' voor volledige eetlust).
3. Kruis zo nauwkeurig mogelijk aan wat u op de huidige dag hebt genuttigd als ontbijt, middageten, avondeten en snacks, vergeleken met wat u normaal at voordat u met uw nieuwe therapielijn van uw antikankerbehandeling begon (d.w.z. wat u hebt doorgegeven in de baseline-vragenlijst): 100% vergeleken met de baseline-situatie, 75%, 50%, 25% of 0%
4. Kruis aan welke van de 2 gegeven opties (d.w.z. middageten en avondeten) uw hoofdmaaltijd (of belangrijkste maaltijd) was van die dag
5. Kruis het aantal aanvullende orale voedingssupplementen aan die u hebt genuttigd op de huidige datum, en vermeld het volume (in ml, volume van elk afzonderlijk supplement).
6. Kruis op de lijst van symptomen aan van welke symptomen u eventueel last had op de huidige dag (bijv. misselijkheid, diarree, braken, ...)

Kruis bij al deze vragen het betreffende antwoord aan in het bijbehorende keuzevakje.

Mocht u een keer vergeten de gegevens in te vullen tijdens de studie, sla die dag dan over. U kunt de gegevens weer noteren voor de volgende dag.

Wij waarderen het zeer dat u bereid bent deze dagelijkse vragenlijst in te vullen!

Onderzoeker	Patiëntcode
☐☐	☐☐☐

Invuldatum vragenlijst (vandaag): ☐☐ DD ☐☐ MM ☐☐☐☐ JJJJ

1 Kunt u een indicatie geven van uw eetlust vandaag? Kruis het betreffende vakje aan, met de volgende overweging: Geen eetlust = 1, volledige eetlust = 10

1 ☐ 2 ☐ 3 ☐ 4 ☐ 5 ☐ 6 ☐ 7 ☐ 8 ☐ 9 ☐ 10 ☐

---

2 Kunt u een schatting geven van de grootte van de porties die u vandaag hebt genuttigd tijdens de volgende maaltijden, vergeleken met uw inname tijdens de baseline-situatie (in %)? Kruis de meest nauwkeurige schatting aan:

A Ontbijt:

100% ☐ 75% ☐ 50% ☐ 25% ☐ 0% ☐

B Middageten:

100% ☐ 75% ☐ 50% ☐ 25% ☐ 0% ☐

C Avondeten:

100% ☐ 75% ☐ 50% ☐ 25% ☐ 0% ☐

D Snacks:

100% ☐ 75% ☐ 50% ☐ 25% ☐ 0% ☐

E Welke maaltijd was vandaag uw hoofdmaaltijd (of belangrijkste maaltijd)?

☐ middageten ☐ avondeten

F Hoeveel aanvullende orale voedingssupplementen hebt u vandaag genuttigd?

0 ☐ 1 ☐ 2 ☐ 3 ☐ 4 ☐

Volume van de aanvullende orale voedingssupplementen: .... ml

---

3 Hebt u vandaag last gehad van één of meer van de volgende symptomen? Kruis alle symptomen aan waar u vandaag last van hebt gehad:

☐ Snel een vol gevoel
☐ Misselijkheid
☐ Braken
☐ Smaakveranderingen
☐ Diarree
☐ Verstopping
☐ Mucositis (ontsteking van de slijmvliezen)
☐ Vermoeidheid/asthenie (zwakte)
