# Supplementary material for: Malnutrition prevalence in cancer patients in Belgium: The ONCOCARE study
Source: Support Care Cancer. 2024 Jan 27;32(2):135. doi: 10.1007/s00520-024-08324-6 (PMC10821821; doi:10.1007/s00520-024-08324-6)
Supplement: Supplementary file 2 — Supplementary file2 (PDF 222 KB) [file 520_2024_8324_MOESM2_ESM.pdf]

Investigateur	Code du patient

FRESENIUS KABI caring for life

IMS Health & Quintiles are now IQVIA™

CONFIDENTIEL

QUESTIONNAIRE QUOTIDIEN DU PATIENT

TITRE DE L'ÉTUDE : Étude observationnelle destinée à évaluer la dénutrition chez des patients cancéreux recevant un traitement anticancéreux palliatif ou néo-adjuvant en Belgique Étude OncoCare

CODE DE L'ÉTUDE (N° DU PROTOCOLE) : NuSt-027-CNI, Étude OncoCare

PROMOTEUR : Fresenius Kabi Belgium NV/SA

CENTRE D'ÉTUDE DE RECHERCHE : ____________

INVESTIGATEUR PRINCIPAL : ____________

CODE DE L'INVESTIGATEUR ____________

DATE DE LA REMISE AU/À LA PATIENT(E) PAR LE CENTRE :
À remplir par le centre

Jour __ Mois __ Année ____

DATE DE LA REMISE AU CENTRE PAR LE/LA PATIENT(E) :
À remplir par le centre

Jour __ Mois __ Année ____

Investigateur Code du patient

INSTRUCTIONS

Merci beaucoup pour votre intérêt pour la participation à notre étude !

Comme vous en avez discuté avec votre médecin, nous comptons sur votre collaboration pour remplir ce questionnaire quotidiennement au cours des 4 prochains mois (ou selon les indications de votre médecin).

N'oubliez pas que vos réponses seront traitées avec une stricte confidentialité. Les données que vous rapporterez seront identifiées par le nom de l'investigateur et un code du patient dans le coin supérieur droit de chaque page du questionnaire. Le lien entre votre identité et ce code ne sera accessible qu'au personnel travaillant dans votre hôpital. N'écrivez pas votre nom ou toute autre donnée identificatrice (par ex. initiales, date de naissance, etc.) sur une quelconque partie du questionnaire.

Veuillez rapporter ce questionnaire à votre médecin lors de chaque visite de suivi pendant votre participation à l'étude. Vous pourrez recevoir un nouvel exemplaire du questionnaire lors de vos visites. Vous recevrez des instructions de l'équipe de l'hôpital sur l'utilisation de ce nouvel exemplaire.

Lisez attentivement les questions et consultez votre médecin ou l'équipe déléguée (par ex. personnel infirmier, diététicien) en cas de doutes ou d'inquiétudes.

Nous vous recommandons de remplir le questionnaire le soir, pour pouvoir rapporter des informations exhaustives au sujet du jour actuel.

Afin de remplir le questionnaire, veuillez suivre les étapes ci-dessous :

1. Veuillez renseigner la date du jour actuel.

2. Sur l'échelle fournie, cochez le numéro qui reflète le plus précisément votre appétit pour le jour actuel (sur une échelle de 1 à 10 où « 1 » signifie aucun appétit, et « 10 » un excellent appétit).

3. Cochez ce qui correspond à votre meilleure estimation de ce que vous avez mangé le jour actuel au petit déjeuner, au repas de midi, au repas du soir et lors de vos encas, par rapport à ce que vous mangiez normalement avant de commencer votre nouveau protocole de traitement anticancéreux (c'est-à-dire ce que vous avez rapporté dans le questionnaire de référence) : 100 % par rapport à la période de référence, 75 %, 50 %, 25 % ou 0 %.

4. Dans les 2 options présentées (c'est-à-dire repas de midi ou repas du soir), cochez celle qui correspond à votre repas principal de la journée.

5. Cochez le nombre de compléments alimentaires oraux que vous avez pris à la date actuelle, et rapportez le volume correspondant (en ml, volume de chaque complément individuel).

6. Dans la liste de symptômes présentée, cochez les symptômes ressentis le jour actuel, le cas échéant (par ex. nausées, diarrhée, vomissements,...).

Pour chaque question, marquez d'un « X » votre réponse dans les cases à cocher.

Au cas où vous oublieriez de rapporter les données pour l'une des journées au cours de l'étude, passez ce jour et continuez à partir du jour actuel suivant lorsque vous rapportez les données.

Nous apprécions beaucoup votre dévouement pour remplir ce questionnaire quotidien !!

Investigateur	Code du patient

Date de remplissage du questionnaire (aujourd'hui) : ☐☐ Jour ☐☐ Mois ☐☐☐☐ Année

1 Pouvez-vous indiquer votre appétit aujourd'hui ? Veuillez cocher une case ci-dessous en suivant les conventions suivantes : Aucun appétit = 1, excellent appétit = 10

1 ☐ 2 ☐ 3 ☐ 4 ☐ 5 ☐ 6 ☐ 7 ☐ 8 ☐ 9 ☐ 10 ☐

2 Pouvez-vous estimer la taille des portions que vous avez consommées aujourd'hui pour les repas suivants par rapport à votre consommation pendant la période de référence (en %) ? Veuillez cocher votre estimation la plus exacte :

A Petit-déjeuner :

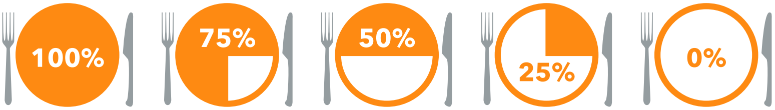

☐ ☐ ☐ ☐ ☐

B Repas de midi :

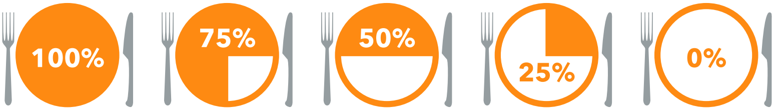

☐ ☐ ☐ ☐ ☐

C Repas du soir :

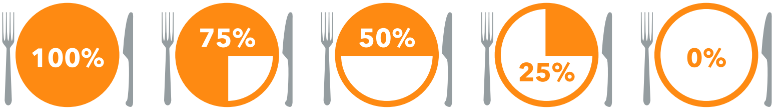

☐ ☐ ☐ ☐ ☐

D Encas :

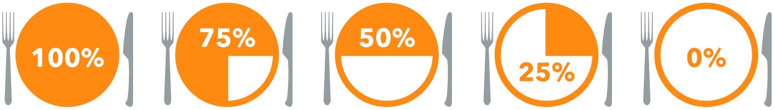

☐ ☐ ☐ ☐ ☐

E Quel repas a été votre repas principal de la journée ? ☐ repas de midi ☐ repas du soir

F Quelle quantité de compléments alimentaires oraux avez-vous consommée aujourd'hui ?

0 ☐ 1 ☐ 2 ☐ 3 ☐ 4 ☐

Volume de compléments alimentaires oraux : .... ml

3 Avez-vous ressenti l'un des symptômes suivants aujourd'hui ? Veuillez cocher tous les symptômes que vous avez ressentis aujourd'hui :

☐ Satiété rapide
☐ Nausées
☐ Vomissements
☐ Altérations du goût
☐ Diarrhée
☐ Constipation
☐ Mucosite (inflammation de la muqueuse)
☐ Fatigue/asthénie (faiblesse)
